# Numerical Assessment of the Efficiency of a New Minimally Invasive Probe for the Isolation of Circulating Tumor Cells

**DOI:** 10.1002/cnm.70032

**Published:** 2025-03-25

**Authors:** Felix Hehnen, Henri Wolff, Sophia Krakowski, Gabi Bondzio, Michael Lommel, Ulrich Kertzscher, Paul Friedrich Geus

**Affiliations:** ^1^ Deutsches Herzzentrum der Charité, Institute of Computer‐Assisted Cardiovascular Medicine Biofluid Mechanics Laboratory Berlin Germany; ^2^ Charité—Universitätsmedizin Berlin, Corporate Member of Freie Universität Berlin and Humboldt‐Universität zu Berlin Institute of Computer‐Assisted Cardiovascular Medicine Berlin Germany; ^3^ Invicol GmbH Berlin Germany

**Keywords:** BMprobe, circulating tumor cells, computational fluid dynamics, in vivo isolation method, liquid biopsy, novel isolation method, numerical analysis, particle tracking, screened blood volume

## Abstract

Liquid biopsy, particularly the isolation of circulating tumor cells (CTCs) from blood, is a promising approach in the fight against cancer. However, the main reason why CTCs are hardly used as biomarkers in the clinic is their complicated isolation from the patient's blood. Existing ex vivo systems use a small volume of blood and can therefore only isolate very few CTCs. To overcome this problem and increase the number of isolated CTCs, a new in vivo method—the BMProbe was introduced, which can isolate CTCs directly from the patient's bloodstream. This study investigates the efficiency of the BMProbe by using Computational Fluid Dynamics simulations to evaluate parameters influencing the attachment probability of CTCs to the probe surface. The analyzed parameters include screened blood volume, residence time, and wall normal rate. Additionally, the impact of probe geometry, vein diameter, and blood flow velocity on probe efficiency was examined. The numerical data suggest that the geometry has a strong influence on cell binding efficiency. Increasing the number of windings from 4 to 32 improves the transport of cells to the surface (negative wall normal rate) from 0 to −29 [mm^2^/s] and the screened blood volume by 138% but decreases the residence time of particles in the close vicinity of the probe by 77%. When compared to experimental data, the screened blood volume and the wall normal rate indicate cell attachment very well, whereas the residence time does not show a significant impact on the attachment of cells. For the 32‐windings BMProbe, the screened blood volume is determined to be 130–313 mL, depending on the vein diameter, which is a multiple of the volume achieved by common CTC isolation techniques.

## Graphical abstract

The CFD study evaluates the efficiency of the BMProbe, a new in vivo method for isolating CTCs from blood. Findings show that increasing the probe's windings increases cell attachment with a screened blood volume significantly higher than that of common methods.

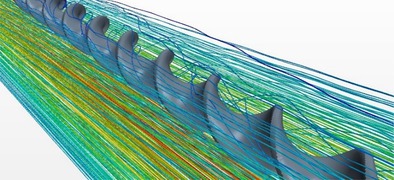

## 1 Introduction

Progress has been made in the diagnosis and treatment of cancer. Nonetheless, it remains one of the deadliest diseases, with nearly 10 million deaths worldwide. Liquid biopsy, the analysis of fluids such as blood and urine, has the potential to improve patient outcomes and reduce mortality. The advantages of liquid biopsy include the low cost and low patient stress for sample acquisition due to its minimally invasive approach [1].

This publication focuses on parameters that have a positive impact on the isolation of circulating tumor cells (CTCs) from the bloodstream. CTCs are cells that have been shed from the primary tumor or metastatic deposits and have entered the bloodstream. Once CTCs extravasate, they hold the potential to form distant metastasis [2, 3]. The analysis of CTCs and their quantification in blood can support physicians in all phases of cancer treatment and diagnosis: For example, the detection of CTCs in blood can help diagnose cancer. This was shown by Xu et al. who included the number of CTCs found in the blood of prostate cancer patients at diagnosis and showed that this greatly improves the detection rate of clinically significant prostate cancers [4]. CTCs can also be used for prognostic evaluation since the CTC count in blood correlates with overall survival and progression‐free survival [5]. Further, the analysis of CTCs enables a treatment stratification and monitoring, for example in lung cancer patients [6, 7, 8]. Also, compared to other analytes that are commonly used in liquid biopsy, such as circulating nucleic acids, proteins, or extracellular vesicles, CTCs are viable, thus allowing further downstream analysis and cultivation of CTCs to develop a treatment plan or study the tumor [9, 10]. A detailed overview of each application is provided in the review by Lin et al. [10].

Although CTCs hold the potential to greatly support physicians in the treatment and diagnosis of cancer patients, the translation into the clinic remains slow to nonexistent since the reliable isolation of CTCs continues to be a technical challenge [1, 11]. This is due to their rarity in blood: 1 mL of blood contains 7.5 × 10^6^ leukocytes, 5–7 × 10^9^ red blood cells, and 2.75–3.2 × 10^8^ platelets [12] and it is assumed that there are less than 10 CTCs/mL in metastasized cancer patients [13]. The low cell count of CTCs is especially problematic for early cancer diagnosis, where the cell count is even lower. Coumans et al. developed a mathematical model which estimates that a 15 times greater blood volume than the current standard of 7.5 mL should be analyzed for CTCs to detect cancer before it can metastasize [14]. A further example is patient‐derived xenograft models where a large number of CTCs (400–1000 cells) are required for successful cultivation [15].

However, most of the developed isolation methods follow an ex vivo approach, which has the challenge of sorting through billions of blood cells and subsequently purifying samples without losing or damaging CTCs [1]. The ex vivo approaches can be categorized into label‐free isolation (e.g., ScreenCell (Paris, France) [16]), antibody‐based isolation (e.g., CellSearch (Menarini Silicon Biosystems, Bologna, Italy) [17]) and in vivo antibody‐based isolation [18]. To separate CTCs from blood, label‐free isolation methods focus on the physical properties of CTCs (e.g., size and deformability). Antibody‐based isolation methods target either CTCs directly (called positive selection) or leukocytes (negative selection) [19]. The CellSearch system is considered the current gold standard of CTC isolation. However, due to the small blood probe volume of 7.5 mL that the gold standard and the alternative ex vivo methods use, they are not able to provide a large cell yield. To improve the translation into the clinic [9, 20], Batool and Yekula et al. thus recommend screening large blood volumes to increase CTC yield [21].

A possibility to screen a larger blood volume through in vivo antibody‐based isolation of CTCs. The company Gilupi GmbH (Potsdam, Germany) introduced the CellCollector [22]: A minimally invasive cylinder‐shaped probe that is coated with anti‐Epithelial cell adhesion molecule (EpCAM) antibodies. The probe is inserted into the median cubital vein, where it isolates passing CTCs during its 30‐min incubation. However, based on a numerical analysis by Dizdar et al. [23], only 0.33–18 mL of blood is screened during the incubation period. Thus, the minimally invasive approach does not screen a far greater volume of blood than ex vivo methods.

To increase the screened blood volume and the subsequent CTC yield, the company Invicol GmbH (Berlin, Germany) developed the BMProbe: A minimally invasive device with a twisted geometry to increase the interaction with blood and the screened blood volume. The functionality of the probe was verified in an ex vivo clinical study where the CTC count from lung cancer patients was compared to the CTC count of healthy controls. 10 mL blood samples were analyzed from each patient, and it was hypothesized that the BMProbe would be able to collect far more cells in vivo due to the larger screened blood volume [18]. A medical device that screens far greater blood volumes and creates improved flow conditions for cell adhesion compared to current methods would thus have a significant impact regarding the translation of CTC analysis into the clinic.

In this publication, using multiple Computational Fluid Dynamics (CFD) simulations, the efficiency of the BMProbe is quantified. In the following, the term efficiency refers to the ability of the probe to isolate CTCs from the bloodstream. Since a direct simulation of cell attachment has many limitations, high computational costs, and is usually associated with inaccuracies, the efficiency in this study is determined indirectly via three parameters that are known to have an influence on cell attachment. These parameters include the screened blood volume, the residence time of cells near the probe's surface and the transport of cells to the probe's surface (negative wall normal rate). Further, the influence that the geometry of the BMProbe, the vein diameter, and the blood flow velocity have on the efficiency of the BMProbe is presented.

## 2 Materials and Methods

**FIGURE 1:**
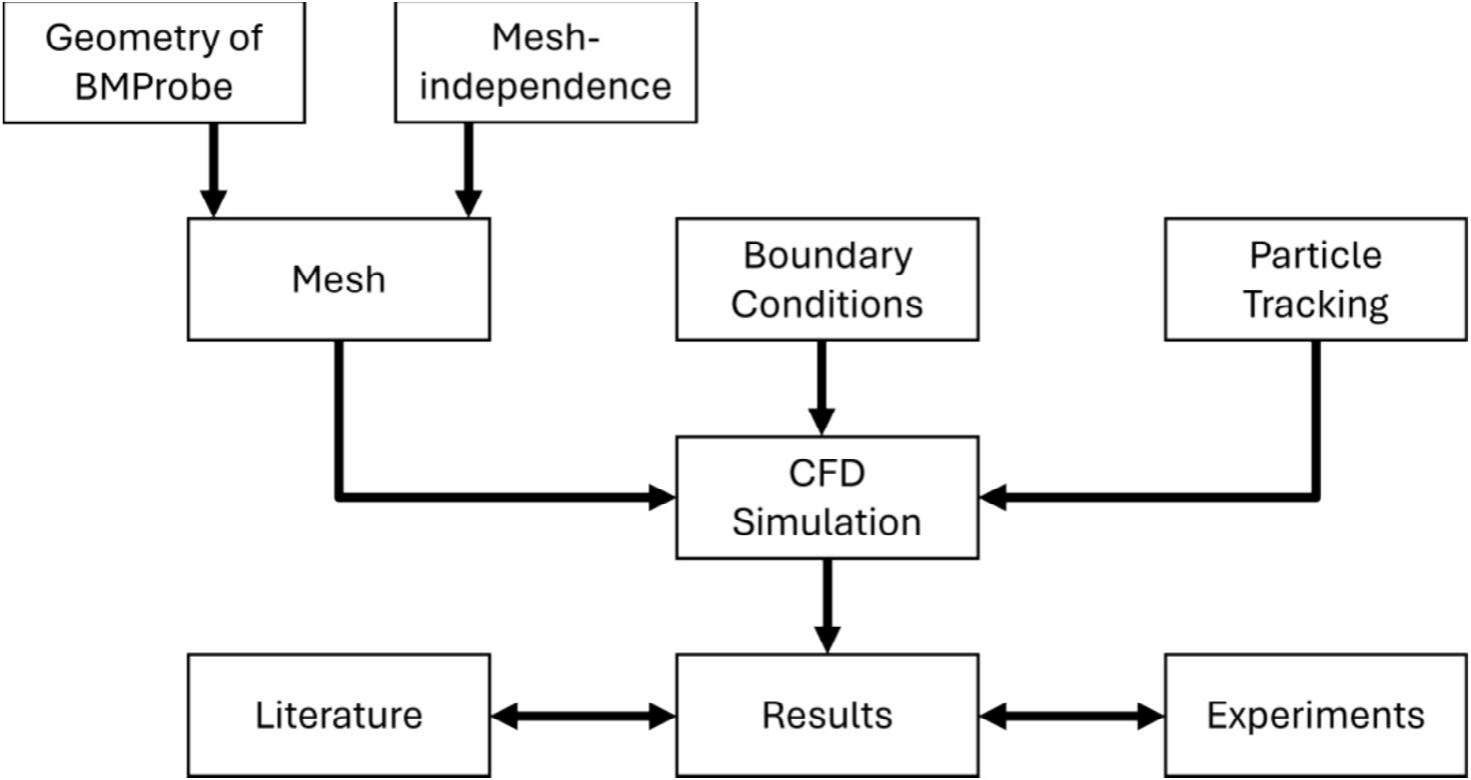
Flowchart of the study design: First, the geometry of the analyzed BMProbe was created and meshed. A mesh independence study helped to select the correct mesh settings. The correct choice of boundary conditions is very important when setting up a CFD study. A Lagrangian particle tracking method was used to determine the screened volume and residence time parameters. After the successful completion of all CFD simulations, the results were determined in a post‐processing step. The results were compared with literature and experimental data.

During the development of the BMProbe, multiple CFD simulations were created that precisely describe the blood flow around the probe. This allowed for the creation of a geometry with improved cell binding efficiency and for understanding how the anatomy and physiology of the individual influence the efficiency. The simulated cases, the cell attachment parameters, and the numerical methods will be introduced. A flowchart of the study can be found in Figure 1.

### 2.1 Description of the BMProbe

**FIGURE 2:**
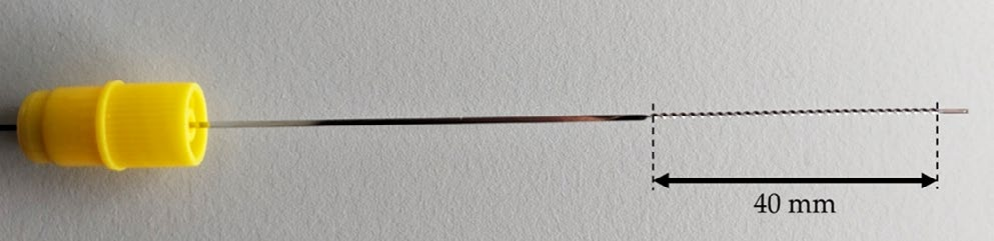
Image of the BMProbe. The probe is inserted through an (18G × 1 1/4”) indwelling cannula into the median cubital vein. It is held in position with the yellow IN‐connector which is screwed onto the cannula. The 32 windings create improved conditions for cell deposition to maximize the cell yield during its 30‐min incubation in the median cubital vein.

The BMProbe, shown in Figure 2, has been previously introduced in detail and is thus only briefly shown here to help understand the performed numerical analysis [18].

The rectangular‐shaped wire, which is then twisted to create the 32 windings, has a width of 0.8 mm, a height of 0.15 mm, and a total length of 160 mm. The number of windings refers to the number of 180° rotations. The 40 mm long section with the windings is called the functionalized part. It is coated with a photoactive polymer to which any Immunoglobulin G antibody can bind, so that specific cells can be targeted during its 30‐min incubation in the median cubital vein. Once the probe is withdrawn, the cells must be washed and fixed. The subsequent downstream analysis can be used to quantify and analyze the cells bound to the surface of the probe. The novel geometry aims to improve cell capture efficiency.

### 2.2 Simulated Cases

**FIGURE 3:**
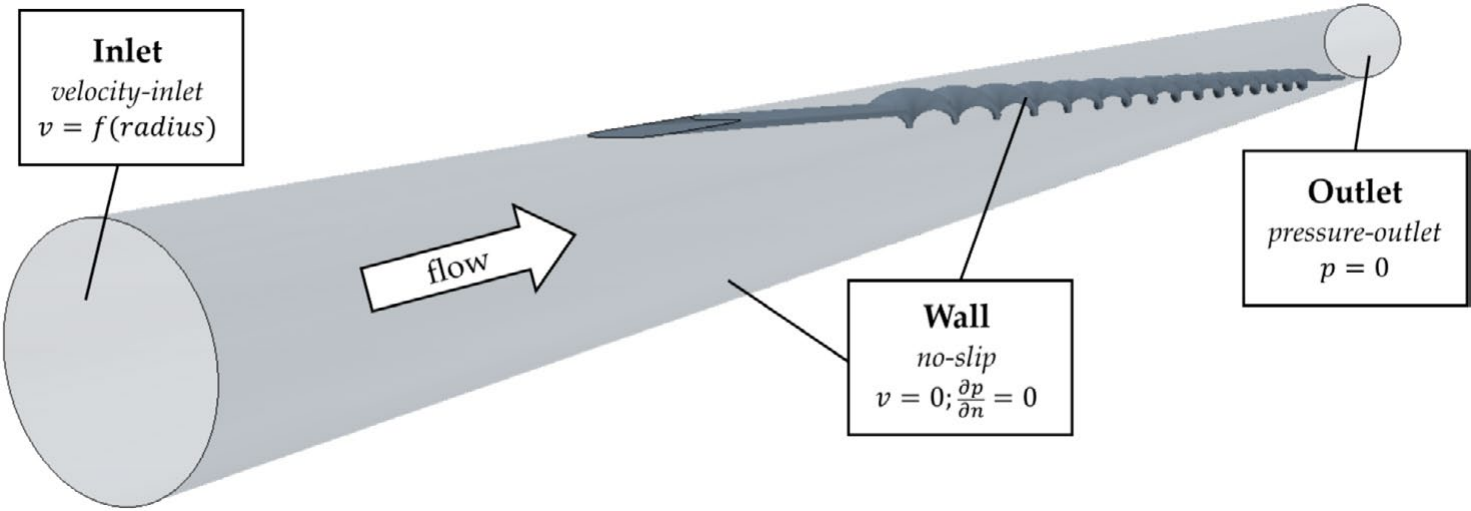
Simulated geometry with boundary conditions. The cubital vein was simplified to a cylinder. The velocity inlet is located on the left‐hand side with a fully developed velocity profile of a pipe flow. The flow passes the probe on its way to the pressure outlet on the right‐hand side. The no‐slip condition applies to the walls of the vein and the BMProbe.

The simulation setup consists of the median cubital vein in which the probe is inserted at an angle of 2.8° (Figure 3). The vein was simplified to a cylinder, based on the anatomical images from Newton et al. [24]. A base case was used as the reference point for this study. Then, simulations were performed where the number of windings of the BMProbe, vein diameter, and mean flow velocity were varied. All variations were compared to the base case.

For the base case, the number of windings was set to 32. This corresponds to the current geometry of the BMProbe. Based on literature, the simulated vein has a diameter of 3 mm and a mean mass flow of 1 g/s [25, 26, 27, 28]. This mass flow rate corresponds to a mean flow velocity of 13.47 cm/s for the base case simulation. All base case settings and variations can be seen in Table 1.

**TABLE 1:** Parameters of the base case and the variations used for the other simulations.

Parameter	Base case	Variations
Number of windings	32	0, 4, 8, 16, 20, 24, 48, 64, 96
Vein diameter [mm]	3	2, 4
Mean flow velocity [cm/s]	13.45	1, 3, 5, 10, 15

Figure 3 shows the position of the probe within the vein. It was chosen to correspond as closely as possible to the planned application. The probe is angled in such a way that its length almost completely fills the diameter of the vein, resulting in an angle of 2.8° between the axis of the vein and the probe. When varying the vein diameter, consequently, the angle of the probe also changes slightly (All angles can be found in Table A1).

### 2.3 CFD Simulation

Flow simulations were performed with STAR‐CCM+ (version 15.02.007, Siemens, Germany). A finite volume formulation was used to discretize the continuity equation (Equation 1) and momentum equation (Navier–Stokes Equation, Equation (2)). The fluid in our study, blood, can be assumed to have a constant density and to be incompressible [29]. Incompressible flows are divergence‐free, which simplifies the continuity equation:

(1) ∇·u=0

To investigate time‐dependent parameters, such as the particle residence time, it is necessary to employ a transient simulation. Consequently, the momentum equation can be expressed as follows:

(2) $$\rho \left(\frac{\partial u}{\partial t}\left(u\cdot \nabla \right)u\right)=-\nabla p+\mu \nabla^{2}u+f$$

In Equations (1) and (2) ∇ is the nabla‐operator (divergence) and u the velocity vector, ρ is the density, p the pressure, μ the dynamic viscosity and f the vector of external forces.

Blood is modeled as an incompressible non‐Newtonian generalized Carreau‐Yasuda fluid (Equation (1), [30]) with dynamic viscosity μ in dependance of the shear rate $\dot{\gamma }$ and with density of $\rho =1050\;\mathrm{kg}/m^{3}$.

(3) $$\mu \left(\dot{\gamma }\right)=\mu_{\infty }+\left(\mu_{0}-\mu_{\infty }\right){\left(1+{\left(\lambda \dot{\gamma }\right)}^{a}\right)}^{\frac{n-1}{a}}$$

With $\mu_{\infty }=0.0035\;\mathrm{Pa}*s$, $\mu_{0}=0.16\;\mathrm{Pa}*s$, λ=8.2s, n=0.2128, and a=0.64, all based on Karimi et al. [30].

Since the highest Reynolds number for all the planned variations of the simulation is 182, we can assume laminar flow for all simulations.

SOLIDWORKS (Version 2018 SP 4.0, Dassault Systèmes, France) was used to create the geometries of the BMProbe and the vein (Figure 3).

#### 2.3.1 Boundary Conditions

The wall of the vein and the surface of the BMProbe were assigned a no‐slip boundary condition. A velocity inlet (Figure 3, left hand side) was used to apply a realistic velocity distribution based on radial coordinates. A fully developed pipe flow was assumed and imposed at the inlet. This ensured that the injected particles followed a physically sensible flow in the inlet region. For the outlet (Figure 3, right hand side) a pressure outlet condition was used (Table A2).

#### 2.3.2 Particle Tracking Method

**FIGURE 4:**
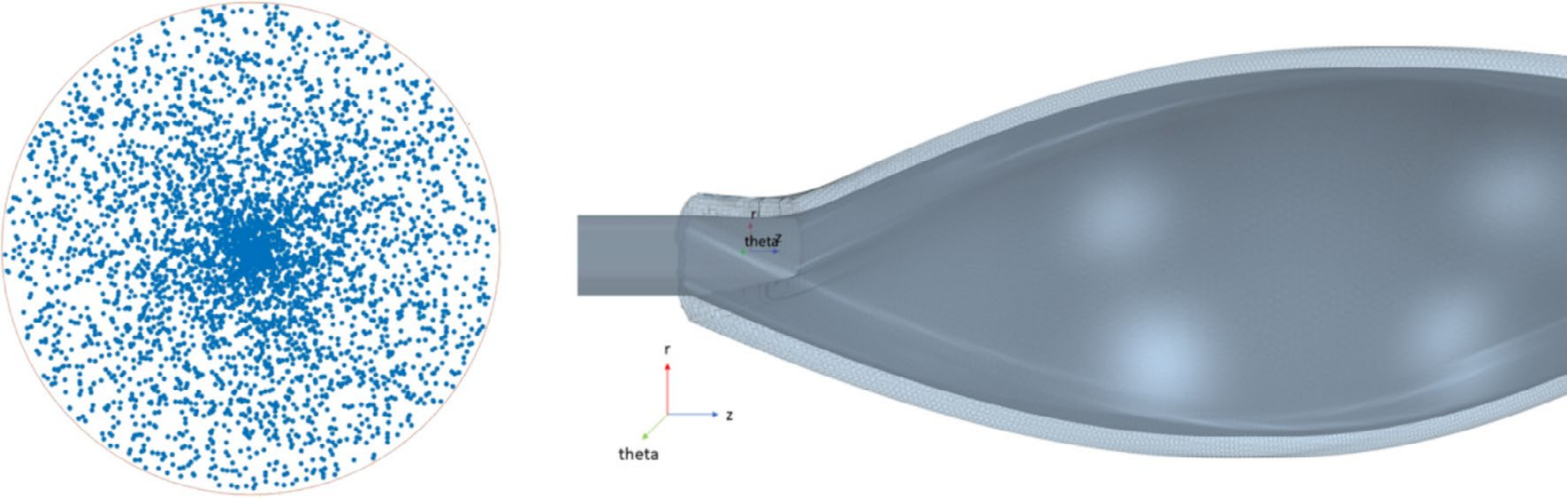
(left) 4000 Particle sources at the inlet are generated to represent a fully developed Hagen‐Poiseuille pipe flow. The particle sources seed 4000 particles per timestep. (right) Part of the BMProbe (dark gray) and the surrounding control volume (light gray/transparent). The control volume only surrounds the functionalized part of the probe. The left part of (right) is the transition to the wire and is therefore not coated.

**FIGURE A1:**
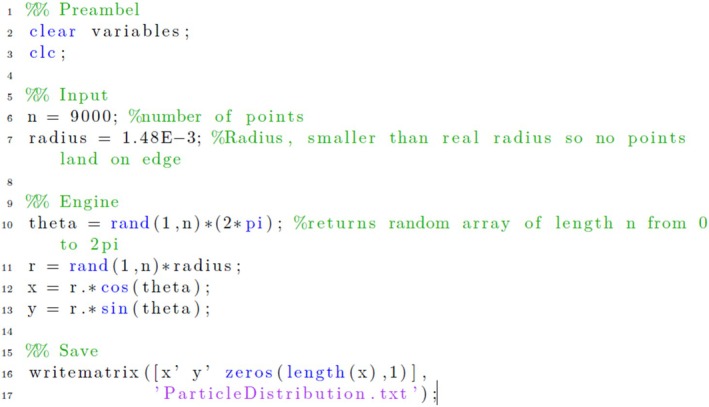
MATLAB code to produce a pseudo‐random distribution of particle sources with a cluster of the sources in the center of the vein. Coordinate points are generated that can be read directly into STAR‐CCM+ to serve as particle sources.

A Lagrangian multiphase model was implemented to enable the simulation of a multiphase flow. Massless particles were selected as a second phase of the flow and injected into the simulation. Since the particles are massless, they will follow the velocity field of the fluid without affecting it. These particles are purely for observation and have no influence on the fluid dynamics. Monitoring their trajectories enabled us to analyze their proximity to the probe (screened blood volume) and their residence time in its vicinity—the so‐called c*ontrol volume*. It is important to mention that these massless particles mimic fluid elements' paths, and do not represent mass‐bearing particles such as cells. The massless particles are not intended to replicate the interaction between CTCs and the probes' surface. The motivation behind injecting particles is solely to replicate the fluid continuum and to track properties such as screened blood volume and residence time of the fluid element within the control volume. This method is not new and has emerged as a powerful tool for estimating residence time in various flow processes. Several studies [31, 32, 33] demonstrate the effectiveness of CFD simulations combined with particle tracking to predict residence time distributions and investigate further flow dynamic properties. Particle injection occurs directly at the inlet. The particle distribution at the inlet was assumed to represent a fully developed pipe flow, known as Hagen‐Poiseuille flow, with faster flow at the center of the pipe compared to the periphery, resulting in higher mass transport there. Consequently, particle sources at the inlet were distributed with a radially decreasing probability of placement. An example of such a distribution is shown in Figure 4 (left). The distribution was implemented using MATLAB (Version R2020a. The script is provided in Figure A1).

4000 particle sources per timestep were chosen because they have been shown to provide a good balance between the accurate simulation of a fluid continuum and a fast convergence time. To determine if and for how long a particle entered the control volume of the BMProbe, passive scalars were used. Passive scalars are quantities that do not influence the flow. In this case, the passive scalar marks the time that a particle spent inside the control volume. This way, the proportion of the screened blood volume and the residence time of particles in the control volume can be monitored.

#### 2.3.3 Control Volume

**FIGURE A2:**
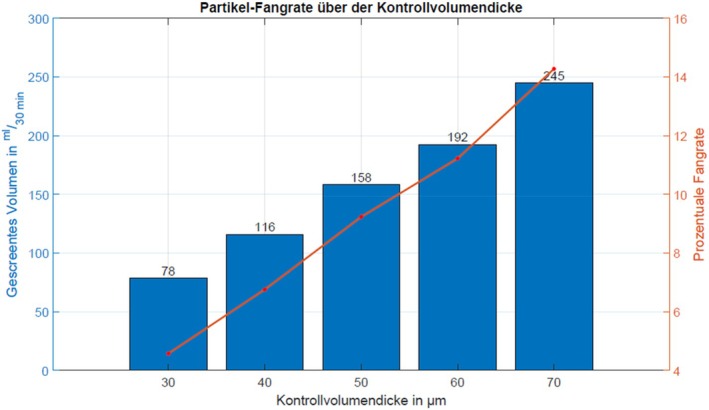
Variation of the Capture distance/thickness of the control volume: A validation study with 5 different capture distances showed an almost linear influence on the screened Volume.

The control volume is a virtual volume around the functional surface of the BMProbe (Figure 4, right). It is assumed that CTCs within this volume form an antigen–antibody reaction with the anti‐EpCAM coating on the surface of the BMProbe. Thus, particles that enter the control volume are considered “screened” The effective capture distance of anti‐EpCAM is not easy to determine and is considered to be highly dependable on the flow rate, shear rate, and antigen density [34]. Dizdar et al. [23] assumed it to be between 25 and 75 μmwhile other publications dealing with the isolation of CTCs using anti‐EpCAM coatings describe similar distances [35, 36, 37]. In addition, we varied the effective capture distance for our simulation from 30 to 70 μm and were able to show that its influence on the screened blood volume is linear (Figure A2). For the sake of simplicity and for better comparability with Dizdar et al., we have decided to assume an effective capture distance of 50 μm for this study.

For the implementation of the control volume in STAR‐CCM+, a scalar was introduced, which marks the cells of the mesh within 50 μm of the functional surface with a scalar value of one. The mesh‐independence study also proved that 5 thin prism layer cells within 50 μm of the control volume were capable of fully resolving boundary layer effects. An increase in boundary layer resolution had no effect on the flow field. Thus, we could rule out errors in the flow field due to incorrect resolution of boundary effects in the control volume.

#### 2.3.4 Mesh

An unstructured polyhedral mesh was used to discretize the geometry shown in Figure 3. Since the region of interest is the flow in the immediate vicinity of the BMProbe, a prism mesh was chosen to refine the mesh in this area. The thickness of the prism layer was set to the control volume thickness of 50 μm. A mesh independence study showed that, with a base size of 100 μm and 5 prism layer cells, a mesh could be generated that would provide a solution that deviates less than 3% from the mesh‐independent solution. More detailed configurations of the mesh can be found in Table A1.

#### 2.3.5 Mesh‐Independence Study

**FIGURE A3:**
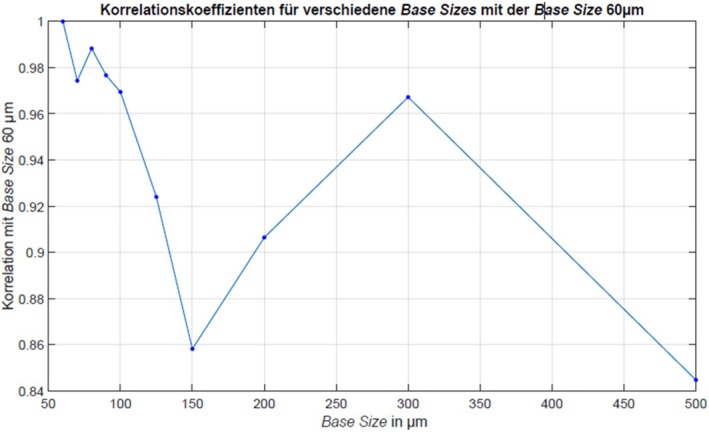
Mesh‐independence study: The correlation of the particle‐entry point in the control volume with the reference mesh (60 μm base size). It is observed that the correlation coefficient begins to converge only at base sizes less than or equal to 100 μm. This implies that the mesh must be very fine to ensure comparability across different simulations. A correlation of 0.97 at a base size of 100 μm provides a good compromise.

Since this work places significant emphasis on massless particles in its evaluation, the particle trajectories of the injected particles are compared for various base sizes. A particle trajectory may consist of hundreds of positions, and each simulation contains thousands of particle trajectories. Comparing all particle positions between simulations would be impractical. Instead, a characteristic point of the particle trajectory is examined more closely: the entry point of the particle into the control volume. These entry points are aggregated into a histogram, which is then established as characteristic for the simulation. Particles that do not enter the control volume during their presence in the computational domain are not considered. Multiple simulations are generated, differing only in their base size. The simulation with the smallest base size, hence the finest mesh, serves as the reference measurement. All other simulations are compared against this reference. The result of this comparison is depicted in Figure A3, showing the PEARSON correlation coefficient, a measure of the linear relationship between histograms, as a function of base size. A coefficient close to one indicates a positive linear relationship. The histogram at a base size of 60 μm serves as the reference.

It is observed that the correlation coefficient begins to converge only at base sizes less than or equal to 100 μm. This implies that the mesh must be very fine to ensure comparability across different simulations. A correlation of 0.97 at a base size of 100 μm provides a good compromise.

### 2.4 Cell Attachment Parameters That Determine the Efficiency of the BMProbe

#### 2.4.1 Screened Blood Volume

The number of particles entering the control volume is measured per time step and divided by the total number of particles injected (4000 per time step). This is called the relative screened blood volume. Particles are counted only once, even if they exit and re‐enter the control volume. It is important to note that the relative screened blood volume converges to a fixed value only after some simulation time, as particles must first be present in the entire computational domain. Therefore, the simulation is evaluated after a few seconds of simulated time, when the relative screened blood volume has converged.

The absolute screened blood volume $V_{S}$ (Equation 4) is calculated from the relative screened blood volume CR, the mass flow $\dot{m}$, the density of the blood ρ and the incubation time t of the BMProbe in the vein. For this study the incubation time is assumed to be 30 min.

(4) $$V_{S}=\frac{\dot{m}*t}{\rho }*\mathrm{CR}$$

#### 2.4.2 Residence Time

The residence time is determined by reading out the passive scalars of the particles. The median was chosen as the representative value of the residence time instead of the mean value. This is because there are a few particles that stay in the control volume for a long time, and therefore the mean value would be highly biased.

#### 2.4.3 Wall Normal Rate

The wall normal rate (WNR, Equation (5)) or wall normal velocity gradient has been shown to be an interesting flow parameter for identifying surfaces where cells are more likely to attach [38]. For near‐wall flows, it describes the velocity gradient that is normal to the wall:

(5) $$\mathrm{WNR}={\left(\frac{\partial v_{⊥}}{\partial x_{⊥}}\right)}_{\text{wall}}$$

The orientation of the WNR is much more significant for the accumulation of cells than its magnitude. A positive WNR describes a flow facing away from the wall, and a negative WNR describes a flow toward the wall. Thus, a negative WNR favors the accumulation of cells at the wall or surface. The implementation of the WNR as a field function for STAR‐CCM+ can be found in the Appendix A.

## 3 Results

The evaluation of the CFD simulations allows assessing the influence of both the geometry (number of windings) and the physiological factors vein diameter and blood flow velocity on the efficiency of the BMProbe.

### 3.1 Influence of the Number of Windings

**FIGURE 5:**
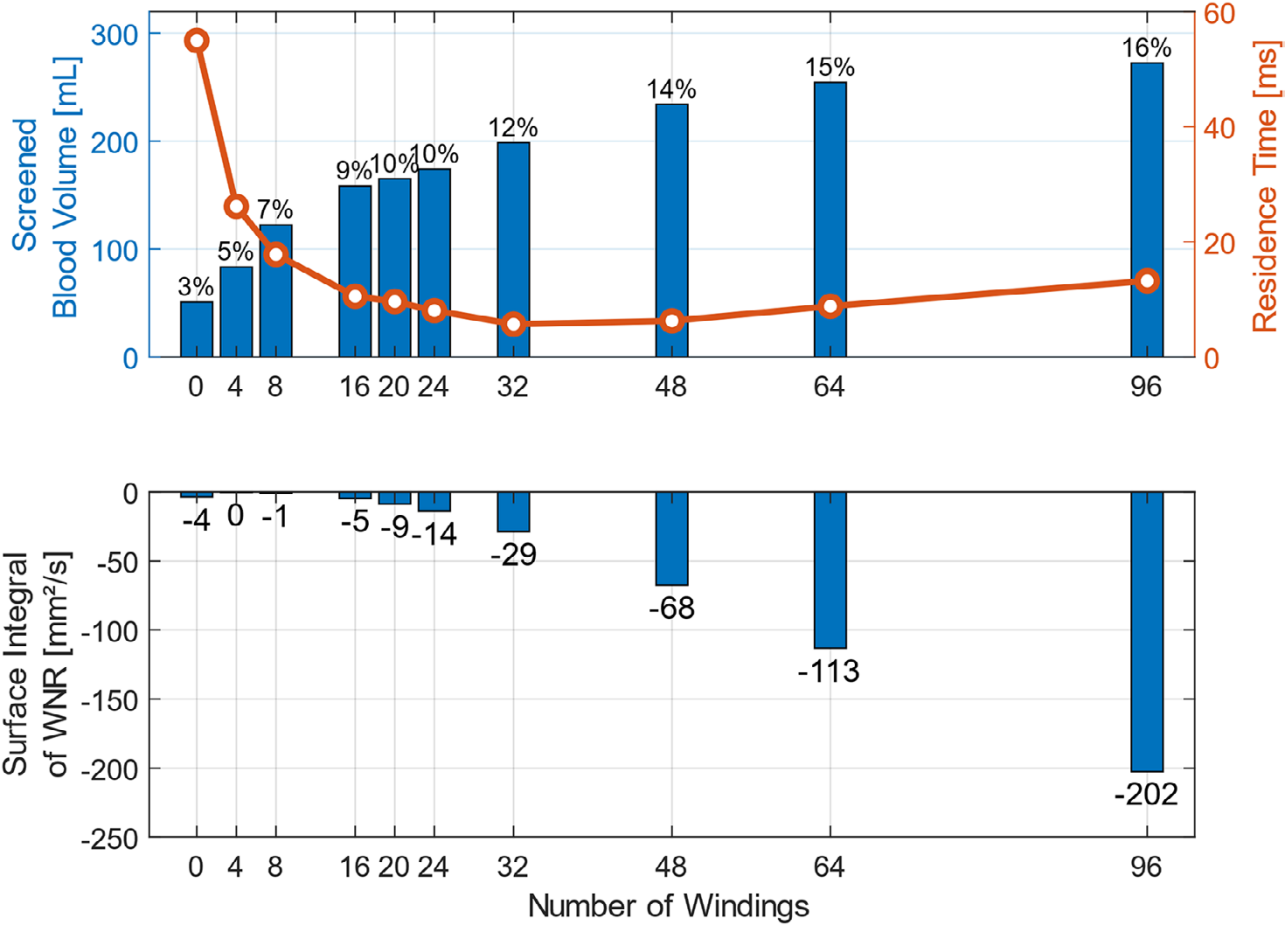
(top) Screened blood volume, (relative values on the tips of the bar plots) and median residence time of the particles depending on the number of windings: A flat steel wire (0 windings) screens significantly less blood with 3% of the total blood flow than a twisted wire. The more windings, the more blood comes into contact with the functional surface coating. Almost 16% of the total blood flow, or 272 mL, can be screened with a 96‐winding BMProbe. Up to 32 windings, the median residence time of the particles decreases significantly and then increases slightly again as the number of windings increases. (bottom) The wall normal rate (WNR) depending on the number of windings: A negative WNR indicates a surface to which cells are transported with the flow to the wall, which therefore favors the deposition of CTCs. The WNR was integrated over the entire functional surface of the BMProbe so that different geometries can be compared. More windings lead to a higher negative integrated WNR.

The influence of the number of windings on the screened blood volume, the residence time, and the WNR was analyzed. Figure 5 shows the screened blood volume in absolute and relative values, the median residence time of particles near the surface of the BMProbe, as well as the integrated WNR depending on the number of windings. A steady increase in the screened blood volume can be observed as the number of windings increases. The influence of the windings on the screened blood volume is clearly recognizable: with an increase from 0 to 4 windings, the screened blood volume increases by 63% from 51 to 83 mL. With 96 windings, the screened blood volume increases to 272 mL, which corresponds to almost 16% of the total blood volume that flows through the vein.

**FIGURE 6:**
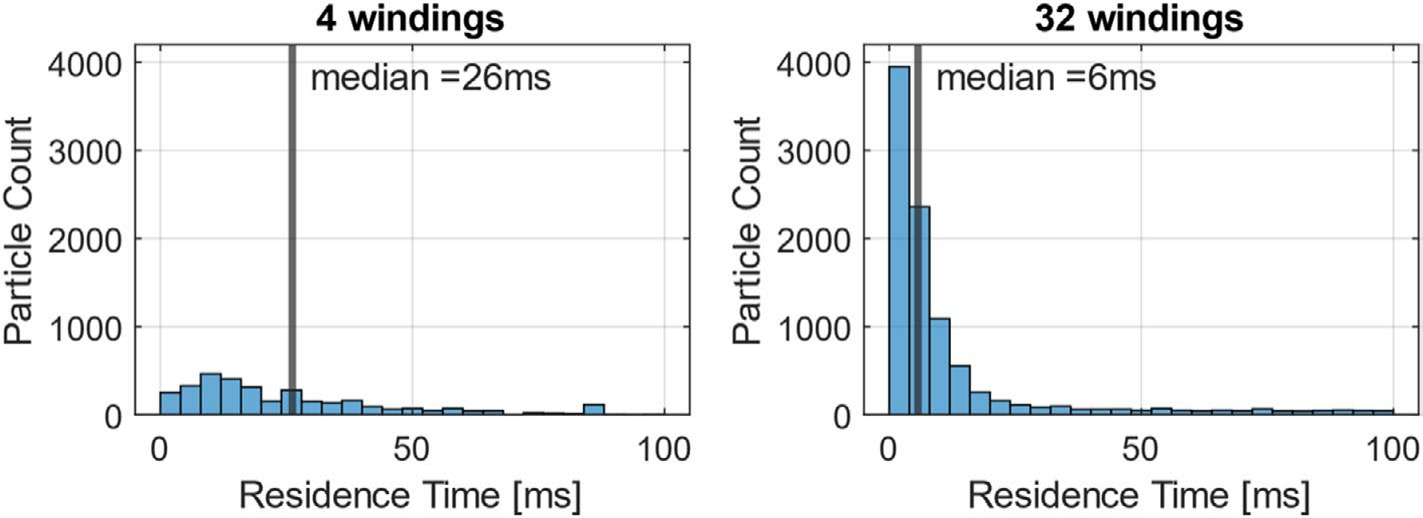
Comparison of the particle residence time distribution of the 4‐winding and 32‐winding BMProbe: For longer residence times above 25 ms, the particle count is in the same range. However, the additional windings lead to an increase in particles that only briefly enter and exit the control volume.

Another favorable parameter for the attachment of cells is their residence time near the functionalized surface. The median residence time of the fluid in the control volume of the BMProbe strongly depends on the number of windings. It can be observed that more windings lead to a shorter median residence time. At first glance, this seems counterintuitive, as it is assumed that the residence time is increased by more windings. This is true for a certain number of particles. The effect is shown for the 4‐winding and the 32‐winding BMProbe in Figure 6: around the same number of particles stays in the control volume for 25 ms or longer. However, the median value is significantly lower for the 32‐winding BMProbe due to the many particles that only briefly enter and exit the control volume. This effect increases as the number of windings increases and reaches its maximum at 32 windings.

Figure 5 (bottom) shows the influence of the number of windings on the integrated wall normal rate (WNR). A negative WNR indicates a surface to which cells are transported from the flow to the wall. It has been shown that cells accumulate significantly more often in areas with negative WNR [38]. For the comparison of different geometries, the WNR has been integrated over the functional surface of the BMProbe. All evaluated geometries have a negative integrated WNR, which is favorable for the accumulation of CTCs. It seems evident that for more than 4 windings, more windings lead to a higher integrated negative WNR.

### 3.2 Vein Diameter

**FIGURE 7:**
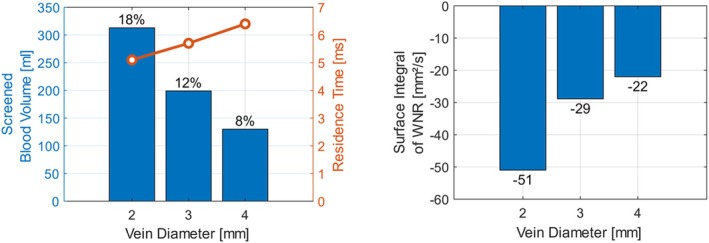
(left) Screened blood volume, (relative values on the tips of the bar plots) and median residence time of the particles depending on the vein diameter: With increasing diameter, less blood passes the BMProbes control volume; thus, the screened blood volume decreases. (right) As the flow velocity also decreases due to a larger diameter, the median residence time increases and the integrated negative WNR decreases.

The influence of the vein diameter on the efficiency of the BMProbe is shown in Figure 7. To ensure that only the influence of the diameter is determined, the mass flow is set to 1 g/s in all cases. This leads to different mean velocities, as mass flow, diameter of the vein, and velocity are in direct proportion to each other. The diameter of the vein has a significant influence on the efficiency of the probe. With a small vein diameter of 2 mm, 313 mL or 18.3% of the blood is screened, while with a large diameter of 4 mm, only 130 mL or 7.6% are screened. This can be explained by the fact that the same amount of blood must flow through a much smaller cross‐section. The BMProbe blocks a larger proportion of the cross‐section than in a vein with a large diameter. The blood must therefore necessarily flow closer to the functional surface of the BMProbe.

The median residence time increases slightly with increasing diameter, as larger veins with the same mass flow lead to slower flow velocities.

The surface integral of the WNR is, for all diameters, negative. The smaller the diameter, the higher the integrated negative WNR. This is also due to the increased flow velocity with small diameters.

### 3.3 Flow Velocity

**FIGURE 8:**
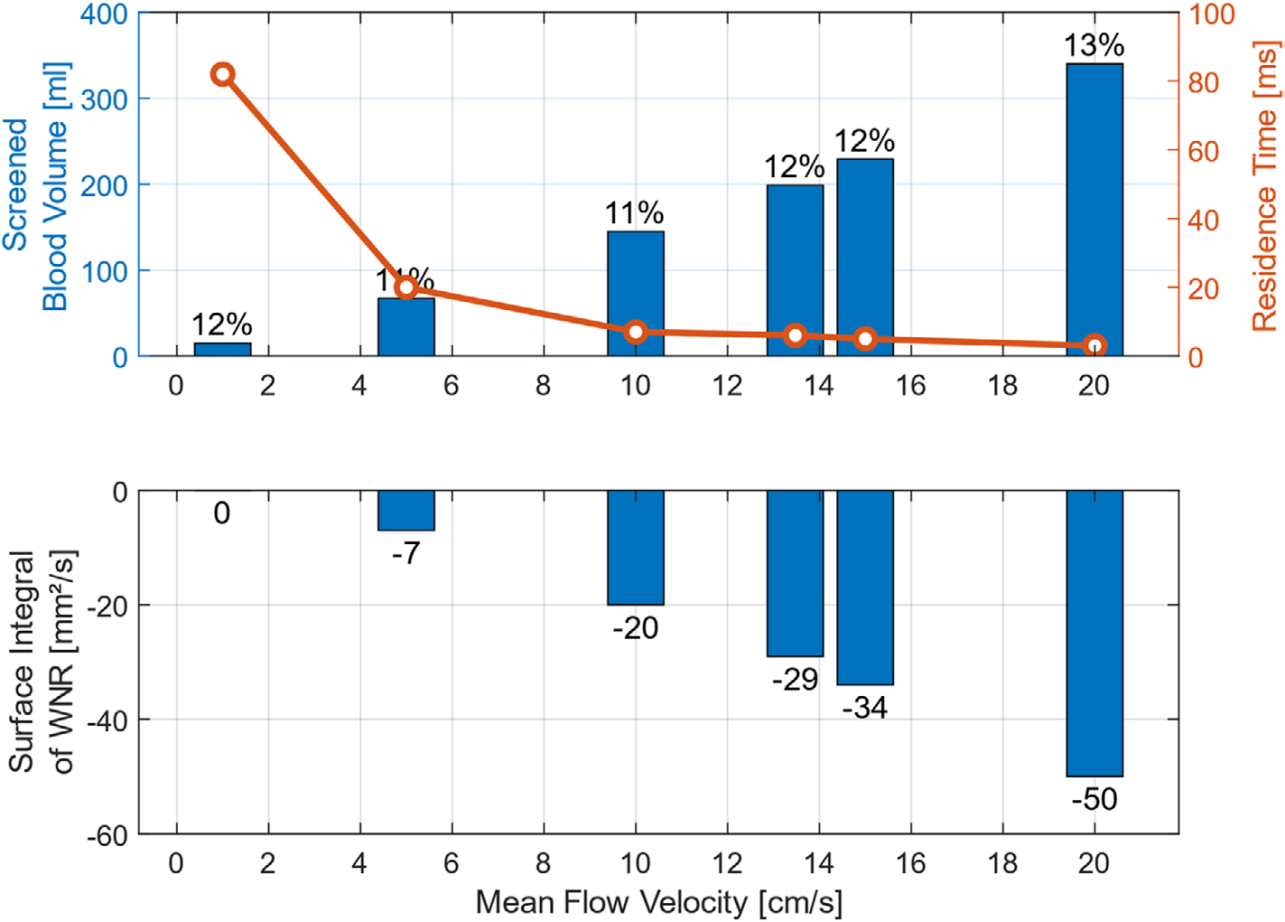
The impact of mean flow velocities on screened blood volume, particle residence time, and WNR: (top) While changes in velocity only affect relative screened blood volume by 1%–2%, absolute volume significantly increases up to 2167% with higher velocities. Particle residence time decreases by 96% with a 20‐fold increase in mean flow velocity. (bottom) Increasing mean flow velocity leads to higher integrated negative WNR, as expected due to higher gradients at the surface of the probe.

The mean flow velocity in the median cubital vein is 13.47 cm/s for the base case; however, depending on the individual, there can be deviations. Thus, Figure 8 (top) shows the screened blood volume and median residence time for different mean flow velocities. A change in velocity has only a small effect on the relative screened blood volume but a strong effect on the absolute screened blood volume. A BMProbe in a vein with a 20 cm/s mean flow velocity can screen 340 mL, or 13.4% of the total vein flow. The same BMProbe in a vein with a 1 cm/s mean flow velocity would only screen 15 mL or 11.6% of the total vein flow. This is 96% less volume in absolute numbers but only 1.8% less in relative numbers.

A change in flow velocity has a large effect on the residence time of the particles. It decreases by 96% while the mean flow velocity increases 20‐fold, as can be seen in Figure 8 (top). Particles that are transported by a slow flow spend a longer time in the control volume.

As can be seen in Figure 8 (bottom), the integrated negative WNR increases with increasing mean flow velocity. This is also to be expected, as higher velocities in the flow field also lead to larger gradients on the surface of the probe.

## 4 Discussion

First, the strengths and limitations of the numerical analysis will be discussed, followed by the discussion of the generated results and a comparison to cell attachment experiments and to the literature.

### 4.1 Strengths and Limitations of the Numerical Analysis

The great strength of this numerical analysis is that it enables a detailed and comprehensive analysis of different geometries and their influence on the cell attachment parameters. This would not have been possible in an experimental framework. For example, as described by Geus et al. [18], it is not possible to manufacture a probe with more than 32 windings that consist of the same material and dimensions as the BMProbe [18]. Using numerical analysis, it was possible to simulate such cases and compare these results to the geometries that are available. Further, cell line experiments are time‐consuming and can lead to a great variation in results, based on the behavior of the cells on a given day, and thus to a misinterpretation of the cell attachment parameters. The numerical analysis allowed greater comparability between the different geometries. Further, using the simulation, it is possible to quantify flow‐based cell attachment parameters in detail.

The particle tracking method used in the numerical analysis considered the particles as screened if they entered the control volume once. It was not analyzed whether they would reenter the control volume at a position further behind in the flow direction. This allowed us to quantify the screened blood volume. However, it neglects the increased likelihood of rescreening a cell at a later position along the surface of the probe.

A limitation of the numerical analysis is the geometry of the vein and the positioning of the BMProbe within that vein. In the simulation, the vein is assumed to be a straight cylinder with a constant diameter. This geometry was chosen as anatomical images from Newton et al. suggest that this is a reasonable approximation of the vein's geometry [24]. Nonetheless, the vein runs through the arm at different heights (distance to skin) this will have an influence on the position of the BMProbe within that vein. In the simulation, the probe is inserted at an angle, similar to the planned in vivo insertion. Depending on the path of the vein within the arm, the probe will be closer to or further away from the wall of the vein than in the simulation. Further, contact with the wall of the vein can lead to bending of the BMProbe, which would influence the geometry of the probe and thus the interaction with the flow. In future applications of the BMProbe, the position within the vein could be analyzed using medical imaging techniques. This data could be used to reevaluate the cell attachment parameters in a new numerical analysis.

Although the flow in the median cubital vein is pulsatile, the flow was set to a constant value in the numerical analysis. To discuss the influence of pulsatile flow, it should be noted that the pulsatility is much lower in veins compared to arteries. The mean ratio of peak to mean flow rate in a cephalic vein (1.36) is significantly smaller than the mean ratio of 3.84 in a brachial artery [39]. In a study of Hehnen et al. [38], experiments were performed with a mean flow velocity between 1 and 5 cm/s, corresponding to a ratio of 5. No significant differences in the number of attached cells could be detected. Therefore, it is not expected that pulsatility in the vein has a significant impact on cell attachment and is thus neglected in this study.

A possible limitation is the selected capture distance of the anti‐EpCAM antibodies on the surface of the probe. The distance of 50 μm was selected based on numerical analysis performed by Dizdar et al. for a similar simulation [23] and on other publications which deal with the isolation of CTCs with anti‐EpCAM antibody [35, 36, 37]. In addition, we were able to show that the screened volume scales linearly with the capture distance in a range of 20–70 μm. If experimental validation of the capture distance from anti‐EpCAM is possible in the future, the results of this study can be adapted accordingly.

### 4.2 Discussion of the Cell Attachment Parameters

#### 4.2.1 Screened Blood Volume

It is essential to stress the difference between the determined screened blood volume and the efficiency of the BMProbe to collect CTCs from the venous bloodstream. As previously described, cell attachment is dependent on multiple parameters that were analyzed in this publication. The screened blood volume only describes how much of the total flow comes into the proximity of the surface of the probe, where an attachment of the cells to the surface is possible due to the antibody coating on the surface of the BMProbe. Mass‐bearing CTCs will follow different flow paths than the massless particles used in this simulation [40, 41]. The parameter screened blood volume must not be equated with the number of screened CTCs. It is important to note that the study's objective was not to simulate the exact behavior of CTCs in the bloodstream, but rather to find parameters with low computational cost that can adequately predict the likelihood of CTC attachment.

#### 4.2.2 Residence Time

A longer residence time means that the cell remains in the proximity of the surface of the BMProbe for a longer time. Thus, the cell follows the geometry of the probe longer, and it is reasonable to assume that it is more likely to attach to the surface of the probe. In the shown results, it is visible that independent of the number of windings, there is a similar number of particles that have a residence time of 20 ms and more. However, with an increasing number of windings, more cells enter the vicinity of the surface of the BMProbe with a very short residence time. Since the experimental results have shown that cell attachment increases with an increase in the number of windings, it could mean that the residence time is negligible for the cell attachment process since there possibly is a near‐instantaneous capturing of cells with the antibody coating. In the literature, the adhesion kinetics of CTCs and anti‐EpCAM have been studied extensively, and it involves complex interactions [42, 43]. However, it has not yet been possible to quantify a correlation between residence time and the number of CTCs bound with anti‐EpCAM. Based on the findings of this study, one could assume that the required residence times are low (< 25 ms). However, further experiments are needed to validate this assumption and determine the duration required for cell isolation.

#### 4.2.3 WNR

Based on the numerical results and a comparison to the experimental data using probes with a different number of windings [18], the WNR has a significant impact on the number of cells that are bound. The parameter was first reported by Hehnen et al. [38] for a specific winding. A high WNR suggests increased convection toward the wall, indicating that cells or particles (such as CTCs) are more likely to be “flushed” against the wall, potentially leading to local accumulation or a higher probability of attachment. In this publication, the WNR was integrated over the entire area of interest for the first time. The surface integral of the WNR quantifies the net transport of cells, particles, or fluid elements toward the wall in the area of interest. It appears to be a useful parameter helping to identify regions prone to accumulation. In addition to the isolation of CTCs, this finding may be highly relevant to other aspects of biofluid mechanics, such as platelet adhesion, cell‐wall interaction, endothelial cell damage, and plaque formation. From a pragmatic standpoint, this could help in the development and optimization of blood‐carrying medical devices or assist in the cultivation of cells on artificial materials.

The numerical analysis has shown that it would be advantageous to increase the number of windings. However, this is not practical. As previously described, the material breaks once a critical number of windings are surpassed. Increasing the temperature of the probe could enhance its formability, potentially allowing for an increase in the number of windings. Additionally, using a wire with a reduced width could be a viable option to create more windings. However, this would result in a decrease in the total surface area, consequently leading to a reduction in the integrated WNR. Even with a different material or fabrication technique, increasing the number of windings is not recommended as it will not be possible to analyze the bound cells under a fluorescent microscope as the surface will be near perpendicular to the field of vision [18]. Finally, it is hypothesized that due to the size and weight of the CTCs, they will not follow the simulated paths once the windings are very close to one another. An interesting approach to further increase the WNR without increasing the number of windings could be the use of porous material for the probe. It has been shown that fluid‐permeable surfaces could increase capture efficiency [36].

### 4.3 Comparison With Experiments

**FIGURE 9:**
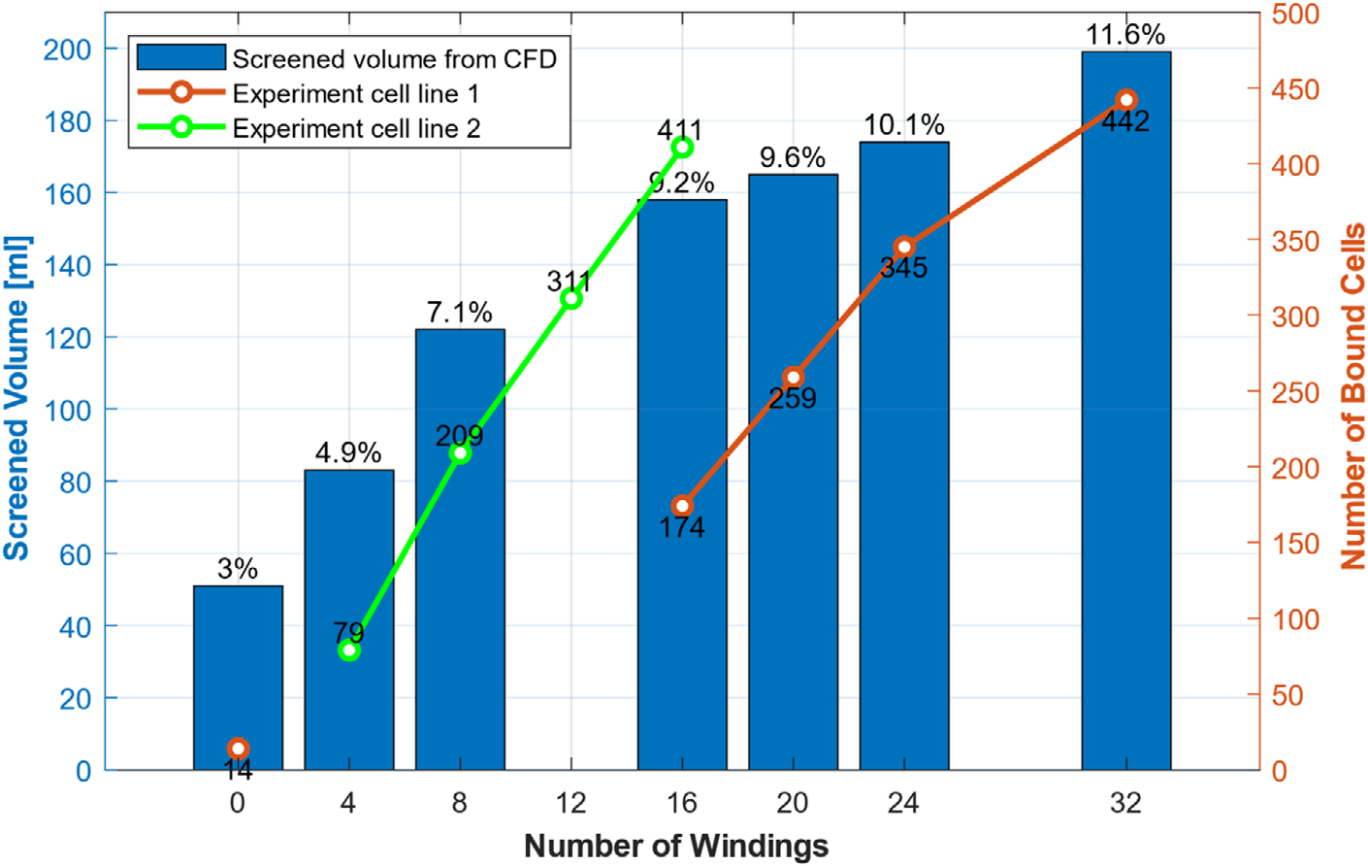
Experimental results from Geus et al. [18] compared to the screened blood volume: Although this figure is no real validation for the simulation, it should put the results into context. The blue bar plot represents the screened blood volume from the CFD simulation. Relative screened blood volumes are indicated on the tips of the bars. The numbers of bound cells from two experiments with different cell lines are shown in orange and green.

The metrics used in this study to evaluate the efficiency of the probe are academic in nature and are derived from a computer simulation. To set the metrics into context, they are compared with cell attachment experiments with the BMProbe. The numbers of attached cells have been taken from a study by Geus et al. [18]. In their study, different numbers of windings were investigated. Two different cell lines were used for this purpose. A clear trend can be recognized for both cell lines used, shown in Figure 9. When compared to the numerically determined parameters, such as the screened blood volume and the WNR, a high correlation can be seen as the number of windings increases. Discrepancies in the trends of the experimental data can be explained by the large variation that usually occurs in experiments with cells from different cell cultures. Further discussion of the experimental results and their potential biases can be found in Geus et al. [18].

### 4.4 Efficiency of the BMProbe

The results show that depending on the vein diameter (2–4 mm), 8%–18% of the total blood volume that passes through the median cubital vein is screened by the BMProbe with 32 windings. For an assumed blood volume of 1800 mL that passes through the median cubital vein during the 30‐min incubation, it can be assumed that 130 mL–313 mL of blood is screened. For patients with veins that are smaller than 2 mm, this value is even higher. This large screened blood volume is up to 42‐fold higher than conventional ex vivo isolation methods [17]. Therefore, the BMProbe fulfills the recommendation of developing isolation methods that screen large blood volumes [21]. Further, the integrated negative WNR is increased with a greater number of windings. There is a 625% increase in integrated negative WNR between a BMProbe configuration that has 0 windings compared to one with 32 windings. The influence of the geometry of the probe is therefore considered to be very high—a fact that is also confirmed by the cell attachment experiments. In addition to the functional coating, the geometry of the probe is a critical factor for its efficiency, on which the developers can have a decisive influence.

Physiological factors such as blood flow and vein diameter are less controllable. Nevertheless, the results of this study show that they can have a similarly strong influence on the screened blood volume of the BMProbe as its geometry. Based on fluid mechanics, higher blood velocity and a smaller vein diameter will always lead to a higher screened blood volume. They also lead to higher integrated WNR; however, the influence is not as strong as that of geometry. Tall people with more blood flow generally also have larger veins than short people with low blood flow. Thus, the influences on the efficiency of the probe are partially balanced out naturally. Therefore, the influence of the geometry on the efficiency of the probe must be considered more significant than the physiological influences.

However, in future in vivo applications of the BMProbe, the influence of the physiology could be used to an advantage. For example, if a patient compresses a small ball with their hand during the incubation, the blood flow in the corresponding median cubital vein will be increased. As the numerical analysis showed, this will significantly increase the efficiency of the probe. Another consequence of this study could be to consider alternative blood vessels for the use of the BMProbe.

### 4.5 The BMProbe in a Clinical Setting

This study has shown that the BMProbe screens a far larger blood volume than current isolation methods and that the geometry of the probe improves the flow conditions for cell adhesion onto the surface of the probe. Therefore, a far greater number of cells are expected to be isolated compared to the current methods.

Since low cell yield is a great limitation of current methods [21], the BMProbe can overcome this limitation and support a translation into the clinic. It is not expected that the minimally invasive approach and 30‐min incubation in the median cubital vein will have any negative ethical implications since studies with a similar product and application, the GILUPI CellCollector, have shown that the minimally invasive approach leads to no adverse events or serious events. In fact, the application was well tolerated [44, 45]. Additionally, any medical device applied in a clinical setting must validate its biocompatibility and safety during regulatory approval.

Regarding the specific application in a clinical setting, the numerical results suggest that the BMProbe is more suited for cancer diagnosis, treatment monitoring, and cell cultivation for treatment than for prognostic evaluation. In prognostic evaluation, the absolute number of CTCs/mL is used to predict overall survival or progression‐free survival, and since the numerical analysis showed the influence of the anatomy (vein diameter) and physiology (blood flow rate) on the attachment parameters, it would mean that the absolute number of bound cells is not comparable to thresholds in the literature. In future, with many in vivo applications, an empirical model could be established based on the weight and height of the individual to determine a personalized threshold value.

## 5 Conclusions

The numerical analysis has shown that the geometry of the BMProbe creates flow conditions that lead to an improved likelihood for cell attachment. This has already been assumed before but was demonstrated quantitatively for the first time in this study. For the 32‐winding BMProbe, the screened blood volume is significantly increased (up to 42‐fold, depending on vein diameter) compared to common ex vivo methods. More windings also lead to a an increased integrated negative WNR, which means that more cells will be transported to the functionalized surface of the BMProbe. Both parameters seem to be useful for evaluating the efficiency of invasive probes to collect the targeted cells. A case can be made that large residence times are not necessary to increase cell yield. However, further analyzes must be performed to validate this.

Physiological parameters such as vein diameter or blood flow also have an impact on the efficiency of the probe. However, utilizing them for CTC isolation will be a future challenge.

When comparing the numerical result to experimental data, it is confirmed that the method used in this study is very suitable for evaluating the probability of cell attachment.

Regarding the translation into the clinic, the numerical study has shown that the BMProbe has the potential to significantly improve cell yield compared to current alternative methods. This is due to the larger screened blood volume and larger surface areas with negative WNR, which positively influence the cell attachment to the surface of the probe. The results of this study and the established methods will be used to further improve the geometry of the BMProbe. One particular success of this study is the effective use of the WNR parameter. This parameter is not limited to CTCs alone but could also be used for other cell wall interactions in the future and thus improve the development of blood‐bearing medical devices.

## Author Contributions

**Felix Hehnen and Paul Friedrich Geus:** conceptualization. **Felix Hehnen and Henri Wolff:** methodology. **Henri Wolff:** software. **Felix Hehnen, Henri Wolff, Sophia Krakowski, Gabi Bondzio, and Paul Friedrich Geus:** validation. **Felix Hehnen and Henri Wolff:** formal analysis. **Felix Hehnen and Henri Wolff:** investigation. **Gabi Bondzio and Ulrich Kertzscher:** resources. **Felix Hehnen and Henri Wolff:** data curation. **Felix Hehnen and Paul Friedrich Geus:** writing – original draft preparation. **Felix Hehnen, Henri Wolff, Sophia Krakowski, Gabi Bondzio, Michael Lommel, Ulrich Kertzscher, and Paul Friedrich Geus:** writing – review and editing. **Felix Hehnen and Henri Wolff:** visualization. **Michael Lommel, Ulrich Kertzscher, and Paul Friedrich Geus:** supervision. **Gabi Bondzio, Ulrich Kertzscher, and Paul Friedrich Geus:** project administration. **Gabi Bondzio, Ulrich Kertzscher, and Paul Friedrich Geus:** funding acquisition. All authors have read and agreed to the published version of the manuscript.

## Ethics Statement

The authors have nothing to report.

## Conflicts of Interest

Paul Friedrich Geus was previously an employee of Invicol GmbH and is still a shareholder of Invicol GmbH and HaimaChek Inc. Gabi Bondzio is an employee and shareholder of Invicol GmbH and HaimaChek Inc.

## Data Availability

The data that support the findings of this study are available from the corresponding author upon reasonable request.

## Appendix A

### Simulation Cases

When the diameter of the vein was varied, the simulation setup changed slightly as well. It had to be ensured that comparable flow conditions and particle inlet conditions exist between the simulations. To keep the flow comparable, the mean velocity at the inlet was kept the same by changing the mass flow antiproportional to the diameter (Table A1). The number of particle sources P was also changed antiproportional to the diameter. The particle source density $\rho_{P}$ was also kept constant with $\rho_{P}=P/A$, resulting in the values for P also given in Table A1.

**TABLE A1:** Simulation Setup for the variation of the vein diameter.

Vein diameter [mm]	2	3	4
Mass flow [g/s]	0.44	1	1.77
Mean flow velocity [cm/s]	13.45	13.45	13.45
Number of particle sources	1778	4000	7111
Angle of the probe in the vein [°]	1.7	2.8	3.5

In addition to these values, the angle at which the probe is positioned in the cylinder was also adjusted so that it does not intersect the wall. The angle is also listed in Table A1.

### Particle Sources

#### Mesh Settings

**TABLE A2:** Mesh settings that differ from default STAR‐CCM+ settings.

Mesh settings	Value
Surface growth rate	1.05
Numbers of prism layer	5
Prism layer stretching	1.3
Prism layer total thickness	50 μm^a^
Volume growth rate	1.05

^a^ Was varied for the investigation of the different control volume thicknesses.

### Implementation of the Wall Normal Rate in STAR‐CCM+

The WNR can be defined as a field function in STAR‐CCM+ with the following input:

$$\begin{array}{c} \mathrm{dot}\left(\$\$\left\{U\_\text{VelocityGrad}\right\}*\$\$\left\{\text{Area}\right\}\left[0\right]+\$\$\left\{V\_\text{VelocityGrad}\right\}\right.\\ {}*\$\$\left\{\text{Area}\right\}\left[1\right]+\$\$\left\{W\_\text{VelocityGrad}\right\}*\$\$\left\{\text{Area}\right\}\left[2\right],\\ \left.\$\$\left\{\text{Area}\right\}\right)/\mathrm{mag}\left(\text{\$\$}\left\{\text{Area}\right\}\right)/\mathrm{mag}\left(\$\$\left\{\text{Area}\right\}\right) \end{array}$$
